# Reducing Variability and Removing Natural Light from Nighttime Satellite Imagery: A Case Study Using the VIIRS DNB

**DOI:** 10.3390/s20113287

**Published:** 2020-06-09

**Authors:** Jacqueline Coesfeld, Theres Kuester, Helga U. Kuechly, Christopher C. M. Kyba

**Affiliations:** 1 GFZ German Research Centre for Geosciences, 14473 Potsdam, Germany; coesfeld@uni-potsdam.de (J.C.); theres.kuester@gfz-potsdam.de (T.K.); helga.kuechly@gfz-potsdam.de (H.U.K.); 2 Leibniz-Institute of Freshwater Ecology and Inland Fisheries, 12587 Berlin, Germany

**Keywords:** airglow, artificial light, calibration, VIIRS DNB, nightlights, remote sensing

## Abstract

Temporal variation of natural light sources such as airglow limits the ability of night light sensors to detect changes in small sources of artificial light (such as villages). This study presents a method for correcting for this effect globally, using the satellite radiance detected from regions without artificial light emissions. We developed a routine to define an approximate grid of locations worldwide that do not have regular light emission. We apply this method with a 5 degree equally spaced global grid (total of 2016 individual locations), using data from the Visible Infrared Imaging Radiometer Suite (VIIRS) Day-Night Band (DNB). This code could easily be adapted for other future global sensors. The correction reduces the standard deviation of data in the Earth Observation Group monthly DNB composites by almost a factor of two. The code and datasets presented here are available under an open license by GFZ Data Services, and are implemented in the Radiance Light Trends web application.

## 1. Introduction

Space-based observations of the visible light emissions of Earth at night are a useful indicator of human activity [1,2]. Near brightly lit infrastructure and especially large cities, the signal of artificial light is much larger than the background natural light (reflected starlight and airglow [3,4]). Away from these bright areas, however, variations in natural light make confident detection of artificial light more challenging, especially for sensors with large ground footprints. This poses a problem for monitoring changes in lighting at regional and national scales (e.g., [5,6]), because small but spatially correlated changes in natural light over large areas can outweigh the signal from lit areas, and most land typically consists of unlit areas (e.g., agricultural lands, forests, lakes [7,8]), even in brightly lit countries. In this article, we present a method for correcting for natural light over large areas, in order to improve the sensitivity of the sensor to faint artificial light sources and changes in sum of lights at large spatial scales. We focus on data from the Visible Infrared Imaging Radiometer Suite Day-Night Band (DNB), but in principle the method could be used for other night light observing sensors.

**Figure 1:**
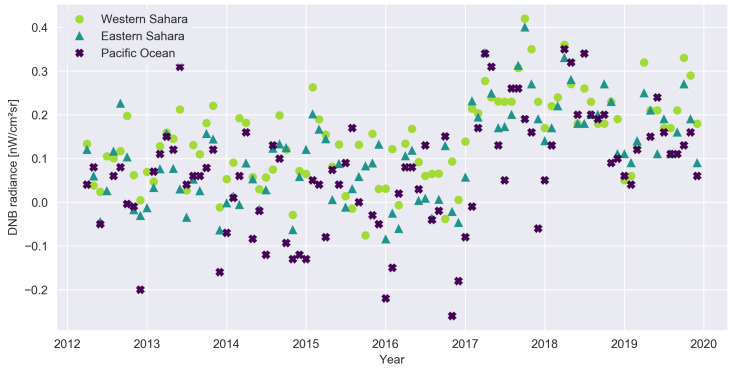
Time series of night light radiance observations for three locations where artificial light emissions are not expected: Western Sahara (23.0875${}^{{}^{\circ}}$ N, 8.525${}^{{}^{\circ}}$ W), Eastern Sahara (21.5458${}^{{}^{\circ}}$ N, 21.4042${}^{{}^{\circ}}$ E), Pacific Ocean (8.8125${}^{{}^{\circ}}$ N, 168.2083${}^{{}^{\circ}}$ E). The jump in 2017 is caused by a change in calibration.

**Figure 2:**
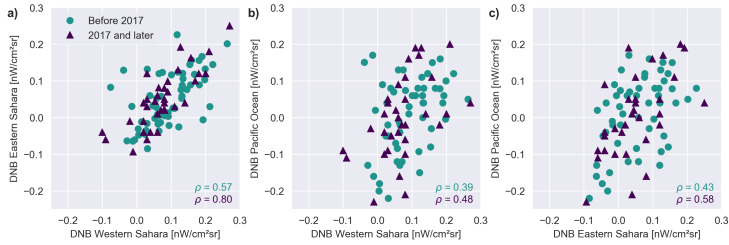
These scatterplots compare the three pairs of data from Figure 1. The symbols indicate the two time periods. For the data after 2017, 0.15 nWcm${}^{-2}\;$sr${}^{-1}\;$was subtracted from the reported radiance, in order to reduce the false appearance of a correlation. (**a**) Eastern and Western Sahara. (**b**) Western Sahara and Pacific Ocean (**c**) Eastern Sahara and Pacific Ocean. The Pearson correlation coefficients (*p*) for each dataset and time period are shown on each figure.

Consider the three light curves shown in Figure 1 for three areas where we do not expect artificial light: the Eastern and Western Sahara and the Pacific Ocean. These data are from the DNB monthly composites produced by the Earth Observation Group [9]. The noticeable increase in all three time series in 2017 is caused by a change in how NASA corrected the data for airglow [10]. Prior to 2017, the ‘zero point’ for DNB includes airglow, so a value of zero would mean no artificial light. Afterwards the zero point should correspond to zero light, and the observation values are therefore nearly always positive (due to natural light). Close inspection reveals a correlation between the data for the two sites in the Sahara, whereas the Pacific data is less correlated with either of the sites in the Sahara. This is shown more directly in Figure 2, where the Pearson correlation coefficients are shown on the figure. Our earlier observation of very high correlations in the ‘noise’ from sites spread throughout the Democratic Republic of the Congo [11] motivated the present study.

Based on these data, we surmise that away from artificial lights, the variation in the ‘noise’ in night light time series arises primarily from changes in the observed radiance of airglow. This variation arises from two factors. First, airglow itself is highly variable. In addition to long-term variations that are related to the solar cycle [12], airglow also changes with the seasons [13], and even on quite short time scales [14]. Second, satellite sensors with a wide swath observe Earth at variable nadir angles $\theta_{n}$. The path length through the glowing part of the atmosphere increases roughly as $1/\cos(\theta_{n})$. This suggests it should be expected to vary up to at least a factor of two, as the DNB images out to nadir angles larger than 60${}^{\circ }$ [15].

The DNB monthly time series produced by the Earth Observation Group (EOG) shown in Figure 1 include only data taken on moon- and cloud-free nights. Moonlight can be bright compared to airglow, and while corrections exist [16], for many applications it is preferable to analyze only data without moonlight rather than potentially introducing noise due to a correction. The number of nights when the moon is up depends on latitude and season, but on average, the moon is below the horizon for about half of the DNB overpasses in any given month. Clouds are more variable, but because of the large spatial scale of weather systems, areas that are separated by distances of less than several hundred kilometers will frequently be imaged on a similar set of nights within a given month. The analysis presented below makes use of these facts to devise a correction for natural light at large spatial scales on moon-free nights.

## 2. Materials and Methods

Our approach is to create a very low spatial resolution image of DNB radiance in areas far from artificial light, and then subtract an interpolated version of this image from the original in order to produce a corrected dataset. This technique requires little data storage, and can be run automatically and rapidly by anyone who has both sets of images. Python scripts for generating the correction images and for reading in and rapidly correcting DNB monthly composites are available in the data and code supplement [17]. The general procedure is as follows: First, we identify locations with little or no artificial light on a world grid. Second, we remove outlier data (e.g., due to wild fires), and interpolate to fill in missing data. Third, we smooth the data within latitude bands. Finally, we expand the low spatial resolution image to the size and projection of the original images, and subtract it from the original to yield a corrected radiance image at full spatial resolution.

### 2.1. Data Sources

The night lights data used in this analysis is the monthly composite image produced by the EOG [9]. We use the dataset produced without the stray light correction, as this procedure introduces noise to some extent, and can leave periodic signal in the images [18]. The EOG images are based on nightly data projected on a 15 arcsecond grid [19], and EOG produces a second file which shows how many individual nights were used to produce the night light image. To reduce the filesize, the Earth is divided into six zones. For one application, we also used an annual composite from EOG, the 2015 annual ‘vcm-orm’ composite image, which “contains cloud-free average radiance values that have undergone an outlier removal process to filter out fires and other ephemeral lights [9].”

To help select areas with little human activity, we used the Global Human Settlement Layer (GHSL) population density map from 2015 [20]. The data are in the same projection as the DNB composites from EOG, but in a 30 arcsecond grid and with a single file for the entire Earth. We therefore resampled the grid to 15 arcseconds (dividing the value in each pixel by 4), and saved the data in 6 files matching the extent and projection of the DNB monthly composites.

### 2.2. Selection of Sites

We created an equally spaced grid over Earth’s surface, covering the extent of the DNB images (from 75° N to 65° S). We did some early experimentation with grid spacings, and found that if the spacing was too small, then there were often points for which we could not find an unlit and uninhabited point nearby. On the other hand, we did not want the spacing to be too large, because it would not capture the variation in background light (particularly the auroral ring, and to a lesser extent the difference on land and sea due to the different albedos [4,21]. We found that a $5^{{}^{\circ}}$ spacing in latitude and longitude worked reasonably well. This equal-angle approach is practical, because it means that the grid points are spaced more closely towards the poles, where the auroral ring is brightest and varies most rapidly. The even steps in latitude and longitude (rather than equal distance) also greatly speed up the processing of the data.

We assigned an equal angle grid that is 72 columns wide and 28 rows tall. This grid of 2016 points ensures the same spacing over latitude in the Northern and Southern hemispheres, it allows us to have an equal number of columns (24) in each of the 6 tiles, and the points also have equal spacing across the equator and the divisions between the longitude tiles. The grid points are inset from the edges of the DNB tiles. The step size in latitude is $S_{\mathrm{lat}}=140^{{}^{\circ}}/28=5^{{}^{\circ}}$, and in longitude is $S_{\mathrm{lon}}=360^{{}^{\circ}}/72=5^{{}^{\circ}}$. The top left point is located at a latitude of $(75^{{}^{\circ}}$ N $-S_{\mathrm{lat}}/2)=72.5{}^{\circ}$ N, and a longitude of $(180^{{}^{\circ}}$ W $-S_{\mathrm{lon}}/2)=177.5{}^{\circ}$ W.

**Figure 3:**
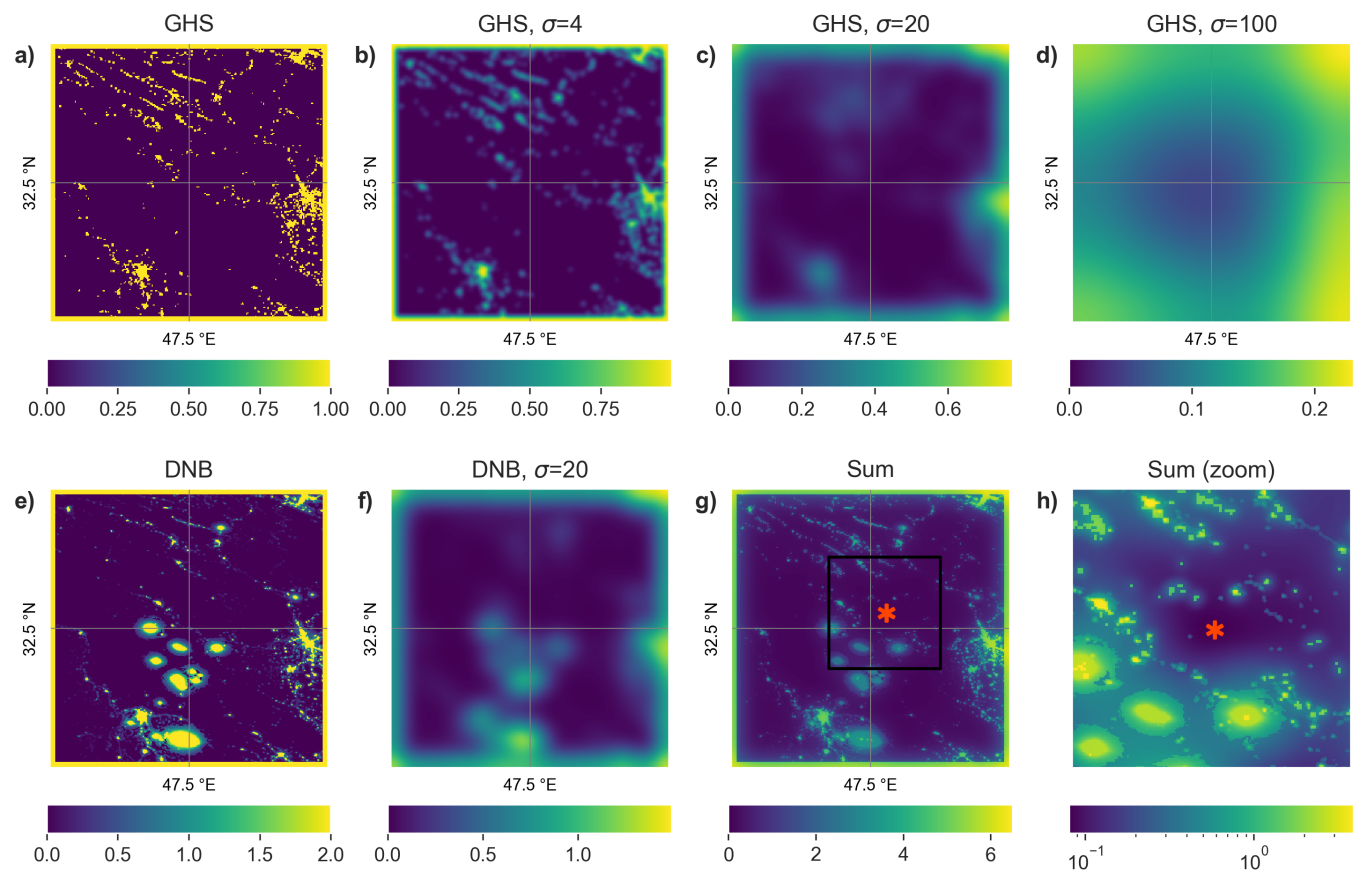
Example of images used to select one site in Iran near oil flaring (32$.5^{{}^{\circ}}$ N, 47.5${}^{{}^{\circ}}$ E). (**a**) Global Human Settlement Layer population density map converted to binary and with a 10 pixel buffer at the edges. (**b**) Panel (**a**) smeared with a Gaussian kernel with width 4 pixels. (**c**) Panel (**a**) smeared with a Gaussian kernel with width 20 pixels. (**d**) Panel (**a**) smeared with a Gaussian kernel with width 100 pixels. The ovals are due to oil flares. (**e**) DNB values (capped at 10 nWcm${}^{-2}\;$sr${}^{-1}\;$and divided by 5) with a 10 pixel buffer at the edges. (**f**) Panel (**e**) smeared with a Gaussian kernel with width 20 pixels. (**g**) Sum of panels **a**–**f**. The red star shows the location of the minimum. (**h**) zoom in of panel (**g**), showing the area near the minimum. Note that the colorbar range is not the same in each panel, and is logarithmic for panel (**h**).

For each of the 2016 grid locations, we searched for a nearby location that was to the greatest extent possible unaffected by human activity (Figure 3). The procedure described below was based to a large extent on trial and error. Other approaches using, for example, different grid spacings or threshold values would likely work just as well.

**Figure 4:**
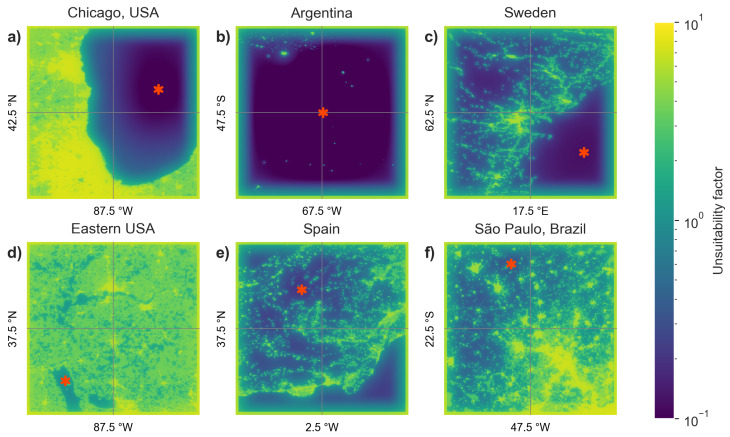
Example of summed images and selected locations for six representative sites. Each image covers an area of 2.083°×2.083°. (**a**) Area near Chicago and Lake Michigan, USA. (**b**) Region of Argentina with few settlements. (**c**) Sweden (top left) and the Gulf of Bothnia (bottom right). Here the buffer at the edges prevents the point from going too close to the edge. (**d**) Highly settled and bright region in the Eastern USA. The selected point is located in the Land Between the Lakes National Recreation Area. (**e**) Densely settled region in Spain. (**f**) Area near São Paulo, Brazil.

For each location, we started by extracting the data in a 500×500 pixel (2.083°×2.083°) area centered from both the GHSL population density map and the 2015 DNB annual composite. This covers most of the area closest to the grid center locations. In cases where the GHSL had no population in the entire 500 × 500 pixel area, we assigned the central pixel as the chosen location (One exception was the point at 62.5° N, 122.5° W, which had a very low density population spread over a large area. We assume this to be treaty land, and used the central location as our point.).When GHSL had data, we converted the 500 × 500 pixel image to a binary image, coded as 1 in areas with population, and 0 in areas without population. We artificially set a value of 1 for all points within 10 pixels of the image edge (see the yellow “frame” around Figure 3a). This prevents the selected point from being too close to an unknown region outside of the selected area (see Figure 4a,c). We then applied a Gaussian filter with three different standard deviations (σ= 4, 20, and 100 pixels) to produce four images (top row of Figure 3).

In order to avoid areas with artificial light emissions away from communities (e.g., oil flares, industrial areas), we used a similar process to the above, using the annual DNB images. As we want to avoid areas with artificial illumination, the total amount of illumination is not particularly important. We therefore capped the maximum value in the DNB image, by setting all values above 10 nWcm${}^{-2}\;$sr${}^{-1}\;$to exactly 10 nWcm${}^{-2}\;$sr${}^{-1}$. (For reference, even villages of only a few hundred people in Europe and North America typically have DNB radiances above 5 nWcm${}^{-2}\;$sr${}^{-1}$. The limit was set to 10 nWcm${}^{-2}\;$sr${}^{-1}$, however, because fluctuations above 5 nWcm${}^{-2}\;$sr${}^{-1}\;$happen with some frequency in the arctic [22].) We then divided the value in all cells by 5, to set the range to [0, 2], and set a value of 2 for all pixels within 10 of the edges to create a frame around the image as before. Next, we applied a Gaussian filter with σ=20 pixels to create a smeared image.

All six images (4 from GHSL, 2 from DNB) were summed, to produce an image with values in the range [0–8], as shown in Figure 3. We then identified the location with the smallest value in this image (red star in Figure 3g,h). The summed images for a selection of six locations around the world are shown in Figure 4.

### 2.3. Assigning Radiance Values to Individual Sites

With a grid of sites selected over the Earth, the next step is to find the radiance at each site in each month, and deal with cases of outliers or missing data. For this we use both the DNB monthly composites and also the associated file showing the number of cloud-free observations for each pixel. We examine all pixels on a 5 × 5 grid centered on the chosen location, and find the median radiance of all pixels that had at least two cloud-free observations. (We have previously shown that the mean of multiple pixels in the DNB dataset is more stable from month to month than is the case for individual pixels [23].) In the next step, this mean is then tested to determine whether it is an outlier (e.g., due to a forest fire).

#### 2.3.1. Outlier Removal and Filling

For each point, we examined the time series from April 2012 to December 2019. In order to account for the shift in NASA’s ‘zero’ value in 2017, for data taken in January 2017 or later we subtract 0.15 nWcm${}^{-2}\;$sr${}^{-1}$. For this time series, we found the 15.9th and 84.1th percentile radiance, and defined half the difference between them as 1σ for that pixel. For each location, we define the outlier threshold as the larger of 1 nWcm${}^{-2}\;$sr${}^{-1}\;$or 4σ plus the median DNB value. Away from auroral lights, the threshold was generally 1 nWcm${}^{-2}\;$sr${}^{-1}$. Pixels with no data (either due to lack of cloud free data or high latitude during summer) were also flagged as outliers.

The outliers were filled using the values from selected grid locations at a similar latitude. For each grid location flagged as an outlier, we examined a 17 × 3 box of grid locations centered on the location we wished to fill. This covers a large swath of longitude (∼1/4 of Earth) but only 10° of latitude. This was done to ensure that regions near the auroral ring will be filled in with data also taken from the ring, while avoiding using these values at lower latitudes. If at least 18 of the 51 nearby grid locations are not outliers, then we find their median, and use that to fill the grid location. This value is not used, however, when filling the values in subsequent outliers.

After completing this process, we noticed occasional larger than normal values in regions with great numbers of lit fishing vessels [24,25]. The positions of fishing areas are not the same from year to year, so a few grid locations occur in places where fishing boats sometimes congregate, leading to occasional outliers. We did not pursue a further correction, since there are few such sites, and they mainly impact sea areas.

#### 2.3.2. Smoothing Data

**Figure 5:**
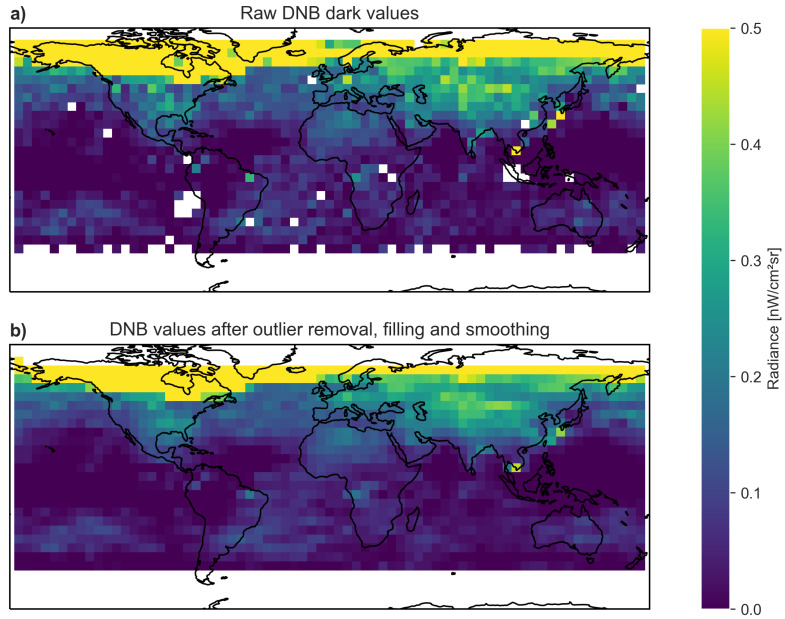
Radiance values at the 2016 grid locations in November, 2015, overlaid on a map of Earth. (**a**) Radiance values in the DNB composite, with missing data shown in white. (**b**) Radiance values after filling outliers and smoothing. Note that the colorbar is clipped at 0.5 nWcm${}^{-2}\;$sr${}^{-1}\;$to show more details. The values in the auroral ring are considerably higher than 0.5 nWcm${}^{-2}\;$sr${}^{-1}$.

Although we used a 5×5 pixel region to find the median radiance for each location, this value could be affected by local environmental factors (e.g., thin cirrus clouds, local snow cover). Since we intend to interpolate between grid location points, we decided to smooth the data using a low-pass filter within each latitude band (again, this is done within a latitude band due to the auroral ring). For each grid location at longitude *i* and latitude *j*, we define the smoothed radiance as $L_{i,j,\mathrm{smoothed}}=\frac{1}{4}\left(L_{i-1,j}+2L_{i,j}+L_{i+1,j}\right)$. Figure 5 shows the radiance values from the monthly composite for November, 2015 before (above) and after outlier filling and smoothing (below). The monthly correction files are saved in csv format, and are available in the data and code supplement [17].

### 2.4. Interpolation

With a global grid of correction factors, the last necessary step is a method to correct any given location on Earth. In our opinion, given the smoothness of the correction factors over most of Earth’s surface, a bilinear interpolation is sufficient. The approach that we found most sensible was to develop a routine that would allow users to correct an entire DNB tile at once. To interpolate across 180° W and to extrapolate to the highest and lowest latitude points, we expand the table of correction factors by one in each direction. The grid points across 180 °W are filled using the corresponding value from the other hemisphere, and the grid points at highest or lowest latitude are filled with a duplicate of the last available value in the regular grid.

We then select the grid points corresponding to the file the user wishes to correct. We treat the grid as an image, and expand the image to match the ground sample locations of the EOG monthly composite file, using the OpenCV-Python routine cv2.resize, with bilinear interpolation [26]. The code next crops the image to match the extent of the EOG monthly composite file. The user can then simply subtract the correction image from the original monthly composite image to obtain a corrected image. The data and code to accomplish this are available in the supplement [17].

## 3. Results

**Figure 6:**
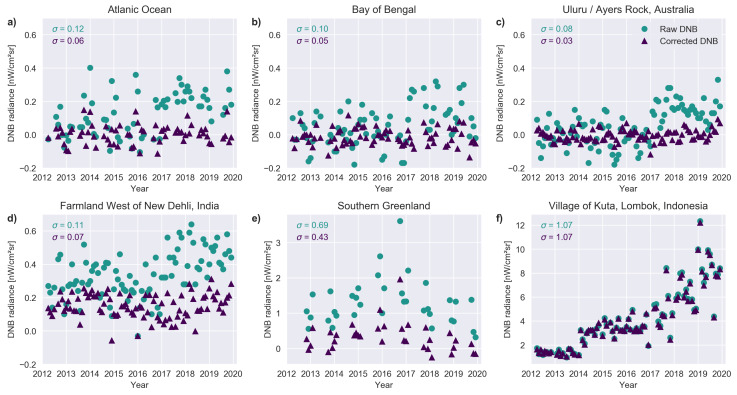
Comparison of corrected and uncorrected time series for a set of individual pixels from the DNB monthly composites. The standard deviation for data taken before 2017 is shown for each dataset (uncorrected top, corrected bottom). (**a**) Pixel located in the Atlantic Ocean (40${}^{{}^{\circ}}$ N, 40${}^{{}^{\circ}}$ W). (**b**) Location in the Bay of Bengal (12${}^{{}^{\circ}}$ N, 88${}^{{}^{\circ}}$ E). (**c**) Uluru, Australia (25.3458${}^{{}^{\circ}}$ S, 131.0375${}^{{}^{\circ}}$ E). (**d**) Area of farmland West of New Dehli, India (28.7417${}^{{}^{\circ}}$ N, 76.3625${}^{{}^{\circ}}$ E). (**e**) Location in southern Greenland (45.45834${}^{{}^{\circ}}$ W, 62.1833${}^{{}^{\circ}}$ N). (**f**) Pixel located in the village of Kuta, Indonesia (8.8911${}^{{}^{\circ}}$ S, 116.2793${}^{{}^{\circ}}$ E). Note that the vertical scale is identical for **a**–**d**, but different for **e** and **f**.

With the correction applied, most unlit pixels around the world should now have a radiance centered on zero. We selected a few locations to demonstrate what the corrected data look like (Figure 6). In all cases, we only considered pixels that had at least 2 cloud free observations during a month. Panels (a) and (b) show ocean locations, where no light is expected. With the correction applied, the values are now centered near zero, and the standard deviation of the data is considerably reduced. Panel (c) shows another unlit location, this time on land, at Uluru, Australia. Again, the scatter is considerably reduced and the data are now centered at zero. Panel (d) shows a location from an agricultural region in Haryana, India. With the correction, it is now possible to see that the satellite sees real light at this location. We believe this light is probably due to skyglow [4] from New Dehli (80 km away) as well as several smaller cities at ∼20 km distance and a few surrounding towns at ∼4 km distance.

A pixel located inside the auroral ring is shown in panel (e) of Figure 6. In this region, DNB detects considerable amount of natural light (c.f. panels a–c). Our approach reduces the values of such regions nearer to zero, and reduces the variation in light considerably. We believe this will be useful for analyses of light from small settlements in the polar regions. Finally, panel (f) shows a pixel centered in a village with considerable (and increasing) artificial light emissions. In such a situation, the impact of the correction is almost negligible.

**Figure 7:**
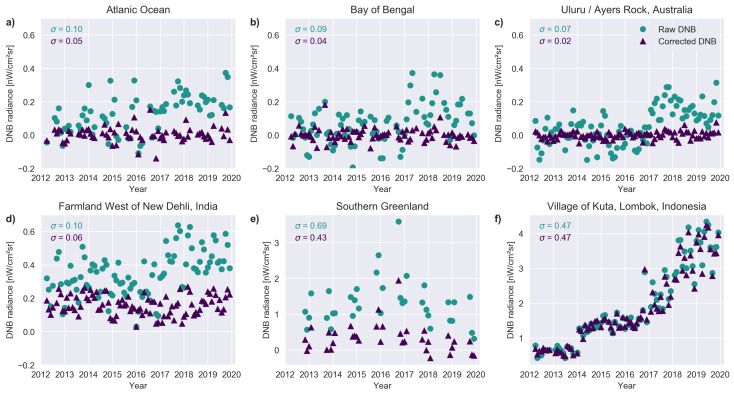
Comparison of corrected and uncorrected time series for the mean of 25 pixels from the DNB monthly composites. The standard deviation for data taken before 2017 is shown for each dataset (uncorrected top, corrected bottom). (**a**) Pixel located in the Atlantic Ocean (40${}^{{}^{\circ}}$ N, 40${}^{{}^{\circ}}$ W). (**b**) Location in the Bay of Bengal (12${}^{{}^{\circ}}$N, 88${}^{{}^{\circ}}$ E). (**c**) Uluru, Australia (25.3458${}^{{}^{\circ}}$ S, 131.0375${}^{{}^{\circ}}$ E). (**d**) Area of farmland West of New Dehli, India (28.7417${}^{{}^{\circ}}$ N, 76.3625${}^{{}^{\circ}}$E). (**e**) Location in southern Greenland (45.45834${}^{{}^{\circ}}$ W, 62.1833${}^{{}^{\circ}}$ N). (**f**) Pixel located in the village of Kuta, Indonesia (8.8911${}^{{}^{\circ}}$ S, 116.2793${}^{{}^{\circ}}$ E). Note that the vertical scale is identical for **a**–**d**, but different for **e** and **f**.

In Figure 7, we show the same set of locations, but this time looking at the average of the center pixel and the 25 surrounding pixels in a 5×5 formation centered on the pixel in Figure 6. The results are similar. As in Coesfeld et al. [23], the scatter in the time series is further reduced compared to when only a single pixel is examined.

**Figure 8:**
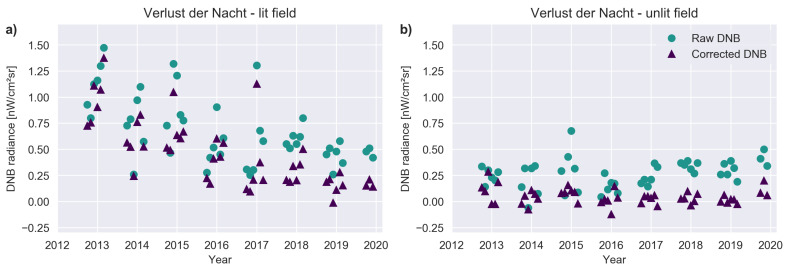
Comparison of corrected and uncorrected time series at the “Verlust der Nacht” biological field site. In both cases the standard monthly composites are shown as circles, the corrected values as triangles. (**a**) Radiance observed in the DNB composite pixel which includes the lit field site (52.6905${}^{{}^{\circ}}$ N, 12.4551${}^{{}^{\circ}}$ E). (**b**) Radiance observed two pixels to the right in the monthly composites (roughly the location of the unlit field site).

We also examined the light trends at a pair of biological field sites in rural Germany (Figure 8). One of the field sites is lit with 12 street lamps, the other is unlit [27]. The two experimental fields are separated by about 620 m (which is smaller than the ground footprint of a DNB observation). Before 17 July 2015, the lit site was illuminated with 70° W High Pressure Sodium lamps (HPS), and afterwards with white LEDs. After applying our correction, the unlit field site has a value close to zero, while the lit field site has a small, positive value in every month. The change in radiance observed by DNB after conversion from HPS to LED in 2015 becomes obvious once the correction is applied. These time series nicely demonstrate the sensitivity of DNB to even very modest numbers of lamps.

## 4. Discussion

Atmospheric airglow is a persistent background of variable strength in night lights imagery. While this can be ignored in some applications (e.g., impacts of war [28,29] or other disasters [30,31]), for other analyses it is a crucial factor. One of the most important examples is in observing trends in light emissions at national [5,6] and international [6,32] scale. These studies often use the “sum of lights” in a country, with the implicit assumption that all observed light is artificial. If each pixel in the country experiences a shift in the same direction due to airglow, the sum of these shifts can rival or exceed the sum of artificial lights. This is especially the case in countries with little lighting infrastructure (e.g., Sierra Leone), high latitudes (e.g., Sweden), or very large areas (e.g., Canada).

**Figure 9:**
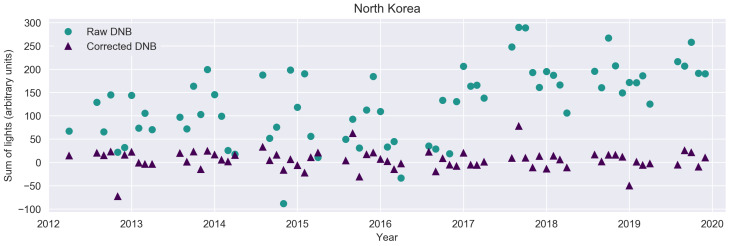
Sum of lights from North Korea. Corrected values are shown as triangles, uncorrected values as circles.

As a demonstration of this, consider the light emissions of the Democratic People’s Republic of Korea (i.e., North Korea) shown in Figure 9. The uncorrected data have considerable scatter, and the change in the way airglow was handled in 2017 appears as a country-wide increase in light. Both of these factors complicate the effort reduce to measure trends in artificial light emissions. For example, the global analysis of Kyba et al. [6] was restricted to areas lit above 5 nWcm${}^{-2}\;$sr${}^{-1}$.

The corrected time series will therefore benefit other research areas that use sum of lights (e.g., economics [33,34]), and this is especially the case for the study of light pollution. For example, models of artificial sky brightness that use DNB data to identify light sources rely on cuts to remove airglow data [35], and these cuts can eliminate the lights of small villages. As another example, Falchi et al. [36] recently examined regional per capita light emissions, and found that the Nordic countries and Alaska had among the highest emissions. Since these regions are strongly affected by the aurora, it would be interesting to see a re-analysis in which the airglow and aurora were removed. Finally, the improved time series will greatly benefit researchers looking at changes in light emission in faintly lit areas with smaller scales (e.g., villages, certified international Dark Sky Places [37], or individual remote lit objects [38]).

Researchers that use the corrected time series should keep two aspects in mind. First, some of the airglow is reflected back towards space from the ground, and this makes land areas (especially those with high albedo) brighter than sea or lake areas [4,21]. This is not a large effect, but it will have an impact on the correction in coastal areas. Second, in wealthy regions with high population densities (e.g., the East Coast of the USA), even areas away from cities are affected by skyglow [35], similar to the case in Panel (d) in Figure 6 and Figure 7. Our correction will therefore remove this effect to some degree. We think that this will be beneficial for most analyses, but some researchers may not wish this artificial light to be removed. This would especially be the case for anyone who wished to remotely sense skyglow from space [4].

Jurij Stare has implemented the correction presented here into the Radiance Light Trends (RLT) web application [39]. For reasons of performance, the RLT webapp does not do a full bilinear interpolation for each pixel. Instead, the 2016 pixel image is expanded by a factor of 16 (4 times in each dimension) using bilinear interpolation, and then nearest neighbor interpolation is used to correct the individual pixels. Readers are invited to explore the data at https://lighttrends.lightpollutionmap.info/. When selecting the "Satellite" from the dropdown menu at the RLT webapp, users can switch between “VIIRS DNB” and “VIIRS DNB - zero correction” to examine the differences between the two time series. As new EOG files are published, new correction files will become available at: https://lighttrends.lightpollutionmap.info/VIIRS_DNB_zero_correction_csv.zip.

## 5. Conclusions

The technique presented here demonstrates that space-based observations of airglow in areas away from city lights can be effectively used to reduce the offset and variability this introduces into night light time series. Our code to correct the DNB monthly composites from the Earth Observation Group and the necessary data are published as a supplement [17].

Remote sensing using visible band data is more challenging during the night than during the day. In dimly lit or faintly lit areas, variation in airglow is the dominant source of variation in observations of the same location at different dates. However, the changes in airglow have little impact in artificially lit urban areas. In such places, most of the observed variation from night to night is caused by changes in the positioning of satellite pixels from overpass to overpass, as well as changes in which lights can be seen in different observation directions [2,15,23,40]. Improved understanding of these type of systematic effects will further increase the value of night lights data for remote sensing of human activities.

## Abbreviations

The following abbreviations are used in this manuscript:

csv	comma-separated values
DNB	Visible Infrared Imaging Radiometer Suite Day/Night Band
EOG	Earth Observation Group
GHSL	Global Human Settlement Layer
HPS	High pressure sodium
LED	Light emitting diode
RLT	Radiance Light Trends
